# In Vitro Ciliotoxicity and Cytotoxicity Testing of Repeated Chronic Exposure to Topical Nasal Formulations for Safety Studies

**DOI:** 10.3390/pharmaceutics13111750

**Published:** 2021-10-20

**Authors:** Larisa Tratnjek, Nadica Sibinovska, Katja Kristan, Mateja Erdani Kreft

**Affiliations:** 1 Institute of Cell Biology, Faculty of Medicine, University of Ljubljana, Vrazov trg 2, 1000 Ljubljana, Slovenia; larisa.tratnjek@mf.uni-lj.si; 2 Chair of Biopharmaceutics and Pharmacokinetics, Faculty of Pharmacy, University of Ljubljana, Aškerčeva cesta 7, 1000 Ljubljana, Slovenia; sibinovskanadica@gmail.com; 3 Lek Pharmaceuticals, d.d., Sandoz Development Center Slovenia, Verovškova 57, 1526 Ljubljana, Slovenia; 4 Institute of Biochemistry, Faculty of Medicine, University of Ljubljana, Vrazov trg 2, 1000 Ljubljana, Slovenia

**Keywords:** nasal drug formulations, mucociliary clearance, ciliary beat frequency, nasal in vitro model, repeated exposure, ciliotoxicity, cytotoxicity, ultrastructural analysis, drug safety

## Abstract

Certain active drugs and excipients of nasal formulations may impair ciliary function and mucociliary clearance. The ciliary beat frequency (CBF) is a key parameter for determining mucociliary clearance rate, and in vitro assessments of CBF have proven to be accurate and reproducible. Since topical nasal formulations are applied with repeated doses, it is essential to elucidate their chronic, as opposed to acute, effect on mucociliary clearance and nasal mucosa. The aim of this study was to assess for the first time the ciliotoxicity and cytotoxicity of nasal sprays intended for chronic treatment (with repeated doses) using a previously designed set-up for CBF measurements. For 2 weeks, the 3D nasal MucilAir™ in vitro models were treated daily with undiluted or clinically relevant doses of mometasone nasal spray, placebo nasal spray, culture medium, or they were untreated. We demonstrated a dose-dependent and time-dependent (cumulative) effect of the nasal sprays on ciliary activity and cytotoxicity using CBF measurements and ultrastructural analysis, respectively. Our results indicate that repeated administration of clinically relevant doses of mometasone nasal spray is safe for in vivo use, which is in good agreement with a previous clinical study. Overall, our study suggests that such in vitro assays have great potential for topical nasal drug screening.

## 1. Introduction

Intranasal decongestants, corticosteroids, antihistamines, and anticholinergics are commonly used topical nasal drugs that are often suggested as first-line therapy for nasal conditions such as congestion, rhinitis, sinusitis, and related allergic or chronic nasal conditions [1,2,3,4,5,6]. They are administered as lavages, drops, squirt systems, or sprays and are used as repeated-dose treatments for nasal diseases [2,7]. In addition to active drugs, various formulation excipients, such as preservatives and absorption enhancers, are present in topical nasal formulations. However, it is essential that active drugs and excipients, individually or in combination, do not interfere with the integrity of the nasal defence mechanism. The main purpose of nasal mucosa is to form a physical barrier that protects the human body from inhaled foreign particles [8], and not to serve as an administration route for drug formulations. The mechanical barrier against pathogen invasion consists of intracellular junctions, mucus, and mucociliary clearance, which depends on the ciliary activity and mucus production of ciliated and goblet cells, respectively [8]. 

The ciliary beat frequency (CBF) is the main parameter that determines the rate of mucociliary clearance [9]. The current clinical method for measuring CBF uses phase-contrast microscopy and a tissue biopsy obtained by brushing the nasal cavity [10,11,12,13]. CBF measurements have been used as an index of ciliary function to assess the ciliotoxicity of nasal drugs in vitro, which has proven to be an accurate and reproducible technique [3,14,15,16,17,18,19,20]. Standardised human in vitro models [21,22,23,24] are easier to use for routine testing and screening than in vivo or ex vivo models, which have the added disadvantage of interspecies differences [25,26]. Furthermore, clinical trials are not suitable for screening a large number of formulation candidates because they are time-consuming, expensive, and often stressful for patients [27]. Even though in vitro experiments cannot accurately mimic in vivo conditions due to a less pronounced or absent protective mucus layer, in vitro CBF analysis should become routine in the development of nasal drug formulations [14,18,28,29,30].

For example, benzalkonium chloride (BKC), which is used as a preservative in nasal sprays, has shown ciliotoxicity in many in vitro studies [15,16,28,31,32,33,34,35]. Its potentially toxic effect has also been demonstrated by several in vivo studies [36,37,38,39,40], and thus caution is advised when using BKC [41,42]. Nevertheless, the toxic effect of BKC can be neutralised in vitro by dexpanthenol, which is a protective and nurturing compound of nasal sprays [28,43]. Therefore, the excipients and active drugs of topical nasal formulations should be tested in vitro for ciliotoxicity and cytotoxicity individually and as combined in the final nasal formulation. Importantly, as topical nasal formulations are used as repeated doses, the cumulative effect versus the effect of single-dose exposure of the nasal formulation should also be tested. For example, repeated 5-day exposure to BKC in vitro resulted in cumulative and irreversible immediate ciliostasis, whereas this was not the case for other tested preservatives (phenylethyl alcohol) [35]. This indicates that the in vitro method can be used to screen for non-ciliotoxic excipients. 

**Figure 1:**
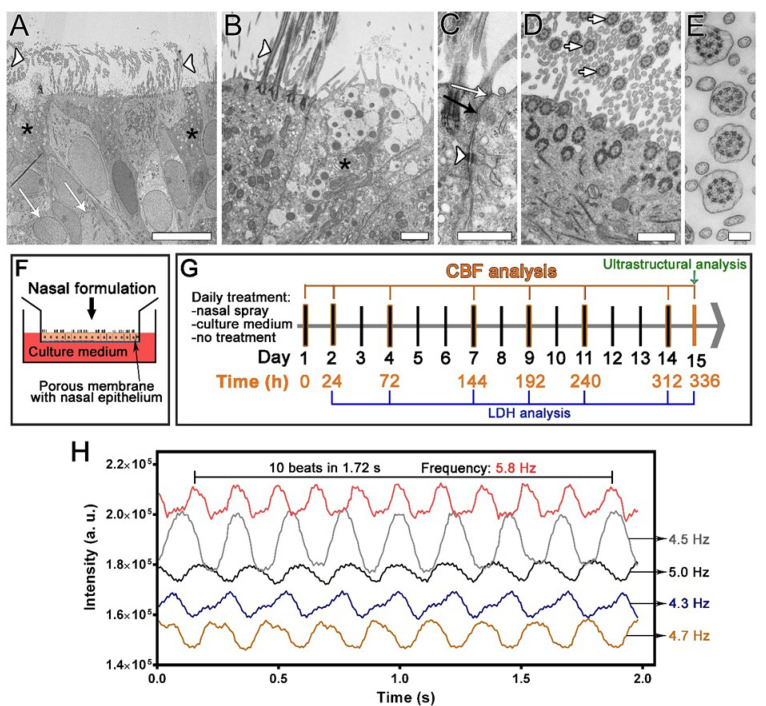
The design of the repeated exposure study utilising the nasal MucilAir™ in vitro models and high-speed digital phase-contrast microscopy imaging. (**A**–**E**) Ultrastructural properties of the nasal in vitro model. (**A**,**B**) A pseudostratified epithelium with ciliated cells (**A**,**B**, white arrowheads indicate cilia), goblet cells (**A**,**B**, black asterisks), and basal cells (**A**, white arrows) is formed. (**C**) Adjacent cells are firmly connected by tight junctions (white arrow), adherent junctions (black arrow), and desmosomes (white arrowhead). (**D**,**E**) Characteristic axonemal structure with 9 × 2 + 2 arrangement of microtubules is viewed in cross section of the cilia (white arrows, **D**,**E**). Bars, 10 µm (**A**), 1 µm (**B**), 600 nm (**C**,**D**), 200 nm (**E**). (**F**) Nasal spray or culture medium was applied to the apical surface of the nasal in vitro models for 30 min daily and then washed off. (**G**) Cells were treated daily for 14 days, and the ciliary beat frequency (CBF) was analysed before the treatments (0 h) and at the indicated times. Ultrastructural analysis was performed at the end of the study. Lactate dehydrogenase (LDH) cytotoxicity assays were performed at the indicated times. (**H**) CBF measurements were performed by analysing the time-dependent greyscale intensity variations resulting from the repetitive cilia beating. Examples of the greyscale waveforms are shown, depicting the change in the grey intensity of the image at 5 different ROI as a function of time. The beat period was determined as the number of beats per unit time, from which the CBF was calculated (one divided by the beat period) and expressed in Hz.

In the present study, we examined the ciliotoxicity and cytotoxicity of repeated doses of the nasal spray formulation. We have previously shown that the combination of the standardised ready-to-use 3D nasal MucilAir™ in vitro model and high-speed phase-contrast microscopy is simple and efficient for single-dose ciliotoxicity studies. A subsequent ultrastructural analysis was performed after the treatment to investigate the cytotoxicity of the nasal spray formulation [34]. The aim of this study was to evaluate the cumulative cilio- and cytotoxicity of repeated doses of nasal spray formulations using a previously developed set-up. During a period of 2 weeks, the nasal MucilAir™ in vitro models were treated daily with undiluted and clinically relevant doses of mometasone nasal spray, placebo nasal spray (without the active drug), and culture medium. Ciliotoxicity was determined with CBF measurements before treatment and then every other day (except on weekends). Cytotoxicity was investigated with the lactate dehydrogenase (LDH) assay every other day (except on weekends) and ultrastructural analysis at the end of the experiment (day 15) (Figure 1). Our results reveal the dose-dependent and time-dependent (cumulative) ciliotoxic and cytotoxic effects of the nasal sprays. The combination of the nasal MucilAir™ in vitro model and high-speed phase-contrast microscopy shows great potential as an in vitro method for the screening and testing of long-term ciliotoxicity and cytotoxicity after repeated doses in the growing field of potential nasal therapies. This could significantly contribute to the three Rs principle of refining, reducing, and replacing the use of laboratory animals. 

## 2. Materials and Methods

### 2.1. Air-Liquid Culturing of the 3D Human Nasal MucilAir™ Epithelium 

The nasal MucilAir™ in vitro models were purchased from Epithelix (Geneva, Switzerland) and originated form a mixture of human nasal epithelial cells collected from 14 healthy donors (cell type hAEC/Nasal, Product Code: EP02, Lot Number: MP0008). Each reconstituted ready-to-use 3D epithelium consists of approximately 500,000 cells. According to the manufacturer’s instructions, the nasal in vitro models were grown on 24-well, 6.5 mm diameter Transwell inserts with 0.4 µm porous membranes (Corning Incorporated, Tewksbury, MA, USA, Cat# 3470). Airway epithelial cell differentiation was triggered on day 6 by removing culture medium from the apical chamber, i.e., air-liquid interface culturing. The nasal MucilAir™ in vitro models were 2 months old upon arrival. They were cultured in a humidified incubator (37 °C, 5% CO_2_) in a proprietary and defined airway culture medium (MucilAir™ culture medium) at the air-liquid interface with culture medium present in the basal chamber only. The medium in the basal chamber (600–700 µL) was replaced every 48 h (except on weekends), and apical wash was performed (with 200 µL of culture medium) to remove the accumulated mucus. The morphology and ciliary activity of the nasal MucilAir™ in vitro models were observed daily under a phase-contrast microscope (DM-IL Leica, Vienna, Austria; objective C Plan 10×/0.22 Ph1 and L40×/0.50 Ph2).

### 2.2. Transmission Electron Microscopy (TEM)

The nasal in vitro models were prepared for TEM as described previously [34]. Fixation of the samples was performed for 3 h at 4 °C with 3% (*w*/*v*) formaldehyde (Sigma-Aldrich, Taufkirchen, Germany) and 3% (*v*/*v*) glutaraldehyde (Serva, Heidelberg, Germany) in 0.1 M cacodylate buffer (pH 7.4). Afterwards, the samples were rinsed overnight at 4 °C in 0.1 M cacodylate buffer. Next, the samples were post-fixed for 1 h at 4 °C in 2% (*w*/*v*) osmium tetroxide (Serva). After short rinse in distilled water, the samples were incubated for 1 h at room temperature (RT) in 2% uranyl acetate (Merck, Darmstadt, Germany) and rinsed again in distilled water. The samples were then dehydrated in a graded series of ethanol baths and embedded in Epon (Serva) by infiltration. Epon polymerisation was performed over the next 5 days with gradual temperature increases (35 °C, 45 °C, 60 °C, 70 °C, and 80 °C) every 24 h. Ultrathin sections (60 nm thick) were then prepared with an ultramicrotome (Leica Microsystems, EM UC6, Vienna, Austria), contrasted with uranyl acetate and lead citrate, and examined at an operation voltage of 80 kV with a transmission electron microscope (CM100, Philips, Eindhoven, The Netherlands) equipped with a CCD camera (AMT, Danvers, MA, USA).

### 2.3. Scanning Electron Microscopy (SEM)

The nasal in vitro models were prepared for SEM as described previously [34]. Fixation of the samples was performed for 2 h at 4 °C with 2% formaldehyde (*w/v*) and 2% glutaraldehyde (*v*/*v*) in 0.2 M cacodylate buffer (pH 7.4) (the fixative was added to both the apical and basal chambers). After fixation, samples were rinsed overnight at 4 °C in 0.2 M cacodylate buffer and then post-fixed for 1 h at 4 °C in 1% (*w/v*) osmium tetroxide. Next, samples at RT were dehydrated in increasing concentrations of ethanol, acetone, and hexamethyldisilazane and then dried. Finally, the samples were sputter-coated with gold and examined at 30 kV with a Tescan Vega3 scanning electron microscope (Brno, Czech Republic).

### 2.4. Treatment with Nasal Formulations

The nasal MucilAir™ in vitro models were incubated daily for 30 min with the (1) nasal spray Mommox^®^/Mometasone Sandoz^®^ (Lek Pharmaceuticals, Ljubljana, Slovenia), containing the active pharmaceutical ingredient mometasone furoate (50 mcg/actuation spray) and the excipients BKC, carboxymethylcellulose sodium, microcrystalline cellulose, sodium citrate, glycerine, citric acid, polysorbate 80, and water for injection; (2) placebo nasal spray without active pharmaceutical ingredients but containing all the above-mentioned excipients; or (3) MucilAir™ culture medium. Intact control cultures were maintained at the air-liquid interface and were not treated with nasal spray or culture medium. For treatment, nasal sprays and culture medium were added to the apical chamber of the nasal in vitro models. Nasal spray application was performed as described previously [34]. The nasal spray pump bottle was shaken, and then the suspension was first applied by spraying into a centrifuge tube and then pipetted into the apical chamber (20 or 40 µL). For the 10-fold diluted concentration, the Mommox^®^ nasal spray and placebo nasal spray were diluted appropriately with culture medium supplemented with 20 mM HEPES. For culture medium-treated nasal in vitro models, MucilAir™ culture medium was pipetted into the apical chamber (20 or 40 µL). The culture medium in the basal chamber (600 µL) was changed with fresh 600 µL of culture medium (on days when CBF was not measured) or HEPES-supplemented (20 mM) culture medium (on days when CBF was measured) in all cultures, i.e., Mommox^®^-treated, placebo-treated, and control cultures (culture medium-treated and intact). The nasal MucilAir™ in vitro models were exposed daily (every 24 h) for 14 consecutive days to undiluted or diluted nasal sprays and culture medium for 30 min at 37 °C in a humidified incubator (5% CO_2_). The nasal sprays were then rinsed off by apical washing of the nasal in vitro models with MucilAir™ culture medium to simulate the removal of nasal sprays from the nasal epithelium by mucociliary clearance in vivo. The culture medium in the basal chamber was changed with fresh medium. The CBF was analysed (as described in Section 2.5) before and 24 h after treatment and then every other day except on weekends (see the scheme of the experiment in Figure 1F–G). During the experiments, the information regarding the used nasal sprays was blinded, i.e., the operator did not know whether mometasone or placebo nasal spray was used. 

### 2.5. CBF Measurement

CBF was determined as described previously [34]. The specific set-up was composed of inverted Leica DM-IL microscope with a phase-contrast objective C Plan 10×/0.22 Ph1, a high-speed recording camera (Basler acA11300-200 µm, Ahrensburg, Germany), the computer connected to a USB3 port and the software Basler (Video recording software V1.1., Ahrensburg, Germany). The nasal in vitro models were first adjusted to RT for 20 min. Recordings of ciliary beating were made in five randomly selected fields of view in each nasal in vitro model. for 1.75–2.5 s with the following settings: exposure time 6.5 ms, video recording rate 143 frames/s. The size of the captured images was 1280 × 1024 pixels. The image sequences were then imported into ImageJ2 [44] using Fiji distribution [45]. On each sequence of frame-by-frame images five square (10–12 pixels) regions of interest (ROI) of different ciliated areas of the nasal in vitro models were selected with high signal-to-noise ratio of the greyscale data obtained from the ROI. Within the ROI of each frame, mean greyscale values were analysed. Areas without apparent cilia beating were not included in the analysis. The total number of analysed ROIs per nasal in vitro model was 25 (*n* = 25). Data were exported to Microsoft Excel (Microsoft, Redmond, WA, USA) in which mean intensity versus time was plotted (Figure 1H). The variation in greyscale intensity in each ROI throughout the sequence of frame-by-frame images resulted from the repeated beating of the cilia. The beat period was determined as the number of beats (complete cycles) per unit of time (Figure 1H), from which the CBF was calculated (one divided by the beat period) and expressed in Hertz (Hz). CBF was measured on 1–2 independent nasal in vitro models, each containing cells from 14 donors, i.e., 14 biological replicates. Results are presented as the relative CBF values, i.e., percentages of the corresponding baseline CBF values measured on day 1 before the treatments (time point 0 h). Measured absolute CBF values ranged between 3 and 8 Hz.

### 2.6. LDH Cytotoxicity Assay

The cytotoxicity of the diluted mometasone nasal spray was investigated by performing an LDH cytotoxicity assay (CyQUANT LDH Cytotoxicity Assay Kit, Invitrogen, Thermo Fisher Scientific, Waltham, MA, USA). Inserts with nasal in vitro models were exposed to 10-fold diluted mometasone or placebo spray or culture medium, as described in Section 2.4. The withdrawn samples (300 µL of culture medium from the basal chamber) on days 2, 4, 7, 9, 11, 14, and 15 were stored at −20 °C until further analysis (see the scheme of the experiment in Figure 1F–G). The LDH cytotoxicity assay was performed according to the manufacturer’s instructions, with minor modifications. Briefly, to obtain samples of maximum and spontaneous LDH activity, the RPMI 2650 cell suspension (≈ 8.3 × 10^5^ cells/mL) was treated for 45 min with 10× Lysis Buffer or sterile ultrapure water, respectively (the samples were prepared in duplicate). After 45 min of incubation at 37 °C, the treated cells were centrifuged at RT and 3000 rpm for 5 min. Next, 50 µL of the supernatant samples (culture medium from the basal chamber of nasal in vitro models treated with mometasone nasal spray, placebo, or culture media or intact nasal in vitro models; spontaneous and maximum LDH activity) were transferred to a 96-well flat-bottom plate in duplicate wells, and 50 µL of reaction mixture was added to each sample well. After 30 min of incubation at RT and protected from light, 50 µL of Stop solution was added to each well, and the absorbance was measured at 490 nm and 680 nm. The LDH activity was determined by subtracting the absorbance value at 680 nm (background) from the absorbance at 490 nm before calculating the % cytotoxicity according to the following equation:

(1) $$\%\;\mathrm{cytotoxicity}=\frac{\mathrm{Compound}˗\mathrm{treated}\;\mathrm{LDH}\;\mathrm{activity}\;-\;\mathrm{Spontaneous}\;\mathrm{LDH}\;\mathrm{activity}}{\mathrm{Maximum}\;\mathrm{LDH}\;\mathrm{activity}\;-\;\mathrm{Spontaneous}\;\mathrm{LDH}\;\mathrm{activity}}\times 100$$

### 2.7. Data Presentation and Statistical Analysis

Data were analysed using the GraphPad Prism software (GraphPad, La Jolla, San Diego, CA, USA). Differences between experimental groups were tested for significance using two-way ANOVA with the Tukey’s multiple comparisons test. Differences were considered statistically significant if *p* ≤ 0.05. All data presented in the graphs are expressed as mean ± S.E. 

## 3. Results

### 3.1. CBF Measurements in the Nasal MucilAir™ In Vitro Model 

The nasal MucilAir™ in vitro models were repeatedly treated to evaluate the chronic ciliotoxicity and cytotoxicity of nasal formulations (Figure 1). Human nasal MucilAir™ cells at the air-liquid interface formed fully differentiated pseudostratified epithelia with three cell types: ciliated, goblet, and basal cells (Figure 1A–E). Ciliated and mucus-secreting goblet cells were homogeneously distributed in the nasal in vitro models. In addition, the nasal in vitro models displayed several other in vivo characteristics such as stratification, mucus production, tight junctions (Figure 1A–E), and ciliary activity, which was analysed by recording ciliary movement with a high-speed digital camera (Supplementary Video S1). The mean (baseline) CBF of the nasal in vitro models was 4.6 ± 0.9 Hz (mean ± standard deviation, *n* = 18 cultures).

### 3.2. Repeated Exposure to Undiluted Nasal Sprays

**Figure 2:**
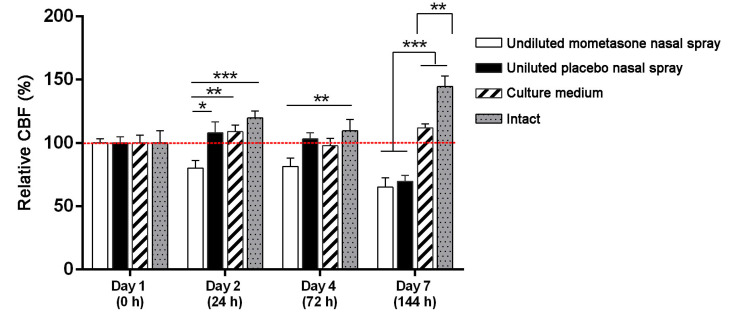
The effect of repeated doses of undiluted mometasone and placebo nasal spray, culture medium or intact condition on the ciliary beat frequency (CBF) of the nasal MucilAir™ in vitro model. Results are presented as relative CBF, expressed as a percentage of the CBF values relative to the corresponding baseline control condition for each tested group measured before treatments. The red dotted line represents 100% (the value of the control condition before treatment). Each bar represents the average relative CBF ± S.E. Mometasone nasal spray inhibits CBF as early as 24 h after the first treatment, and this inhibition persist on days 4 and 7. Placebo nasal spray significantly inhibits CBF on day 7 of continuous treatment. *n* = 25; two-way ANOVA with Tukey’s multiple comparisons test; * *p* < 0.05, ** *p* < 0.01, *** *p* < 0.0001.

**Figure 3:**
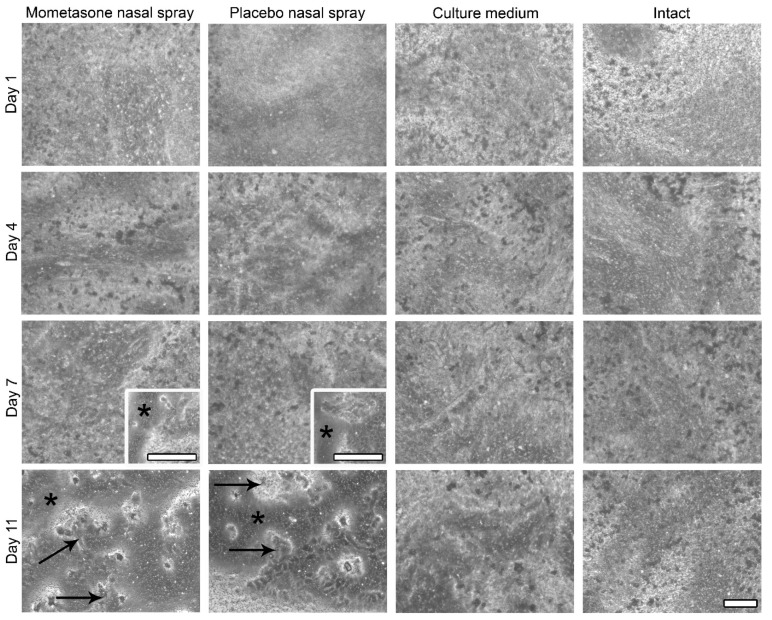
Morphological assessment of the nasal MucilAir™ in vitro models treated repeatedly with undiluted mometasone or placebo nasal spray, culture medium treated or untreated using phase-contrast microscopy. The apical surface view is shown. Cell shedding is observed in the undiluted mometasone- and placebo-treated nasal MucilAir™ in vitro models, which starts at the periphery on day 7 (inserts). Cell detachment is almost complete on day 11. Asterisks indicate areas without cells; arrows indicate the remaining cells on the growth surface. Scale bars, 100 µm.

To investigate the effect of repeated exposure to mometasone nasal spray on CBF, the nasal MucilAir™ in vitro models were initially treated daily with undiluted mometasone and placebo (without the active drug) nasal spray. We have previously shown that single-exposure treatment with undiluted mometasone nasal spray for 3 h increases ciliary activity of nasal MucilAir™ in vitro models by 25.7 ± 2.5% compared to culture medium-treated controls. However, 24 h after shorter (30 min) undiluted mometasone treatment, ciliary activity decreases by 18.5 ± 9.6% compared to culture medium-treated controls [34]. In this study, repeated exposure of the nasal in vitro models to undiluted mometasone nasal spray revealed a cumulative time-dependent effect on CBF. CBF was reduced to 80.2% ± 5.9% and 81.4% ± 6.4% of the baseline CBF (i.e., baseline control condition, which was measured before the treatment at time point 0 h) on days 2 and 4, respectively (Figure 2). After 1 week of daily treatments with undiluted mometasone, the CBF decreased to 65.1% ± 7.4% of the baseline CBF (day 7, Figure 2, Supplementary Videos S2 and S3) and was significantly lower from the culture medium-treated and intact nasal in vitro models (Figure 2, Supplementary Table S1). Ciliostasis was observed on day 9 (Supplementary Video S4). In addition, most cells detached from the growth surface. Namely, cell shedding started on day 7 in the periphery of the cultures and progressed to nearly total cell loss by day 11 (Figure 3, Supplementary Figure S1). On day 11, the experiment was terminated (CBF analysis was performed on days 1–7, Figure 2).

Similarly, daily treatment with placebo nasal spray inhibited the ciliary activity of the nasal in vitro models. A significant CBF reduction was observed on day 7 (to 69.7% ± 4.7% of the baseline CBF, Figure 2, Supplementary Videos S5 and S6), which was significantly lower than the CBF of the culture medium-treated and intact nasal in vitro models (Figure 2, Supplementary Table S1). On day 11, ciliostasis and almost complete cell detachment were observed (Figure 3, Supplementary Figure S1, Supplementary Video S7). As in the mometasone nasal spray-treated cultures, cell shedding started at the periphery on day 7 and progressed to nearly total cell loss by day 11 in placebo-treated cultures (Figure 3, Supplementary Figure S1).

Conversely, daily treatment with culture medium did not affect the ciliary activity of the nasal in vitro models (Figure 2, Supplementary Table S1, Supplementary Videos S8 and S9). No cell detachment was observed (Figure 3, Supplementary Figure S1). The CBF values even increased with time in the intact nasal in vitro models (Figure 2, Supplementary Table S1, Supplementary Videos S10 and S11).

### 3.3. Repeated Exposure to Clinically Relevant Dilutions of Nasal Sprays

**Figure 4:**
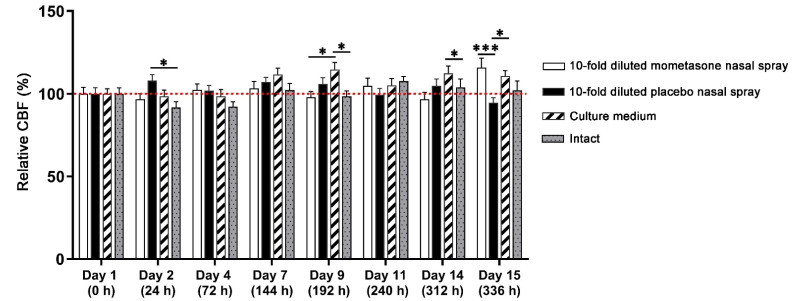
The effect of repeated doses of 10-fold diluted mometasone and placebo nasal spray, culture medium or intact condition on the ciliary beat frequency (CBF) of the nasal MucilAir™ in vitro model. Results are presented as relative CBF values, expressed as a percentage of the CBF values relative to the corresponding baseline control condition for each tested group measured before treatments. The red dotted line represents 100% (the value of the control condition before treatment). Each bar represents average relative CBF ± S.E. Diluted mometasone and placebo nasal spray do not inhibit CBF compared to the culture medium-treated and intact nasal in vitro models. Culture medium treatment increases CBF in the second week of daily treatments. *n* = 50, two-way ANOVA with Tukey’s multiple comparisons test; * *p* < 0.05, *** *p* < 0.001.

Since nasal formulations administered into the nostrils are diluted by mucus under physiological conditions, we tested the effect of repetitive exposure with 10-fold diluted concentrations of the nasal sprays. The dilution factor of 10 has been described as clinically relevant [35,38,46]. Mometasone nasal spray did not impair ciliary activity; no statistical differences compared to the intact nasal MucilAir™ in vitro models were found (Figure 4, Supplementary Table S2, Supplementary Videos S12–S14). Similarly, placebo nasal spray did not impair CBF compared to the intact nasal in vitro models (Figure 4, Supplementary Table S2, Supplementary Videos S15–S17). On day 2, the CBF values in the placebo-treated cultures even increased compared to the intact nasal in vitro models (Figure 4, Supplementary Table S2). Conversely, daily treatment with culture medium increased CBF in the second week of treatments. Namely, CBF increased with time compared to the baseline CBF (time point 0 h). On days 9 and 14, the CBF increased compared to the mometasone-treated and intact nasal in vitro models (Figure 4, Supplementary Table S2, Supplementary Videos S18–S20 (culture medium-treated) and Supplementary Videos S21–S23 (intact nasal in vitro models)). On day 15, the mometasone-treated nasal in vitro models exhibited higher CBF values than those of the placebo- and culture medium-treated nasal in vitro models (Figure 4, Supplementary Table S2).

**Figure 5:**
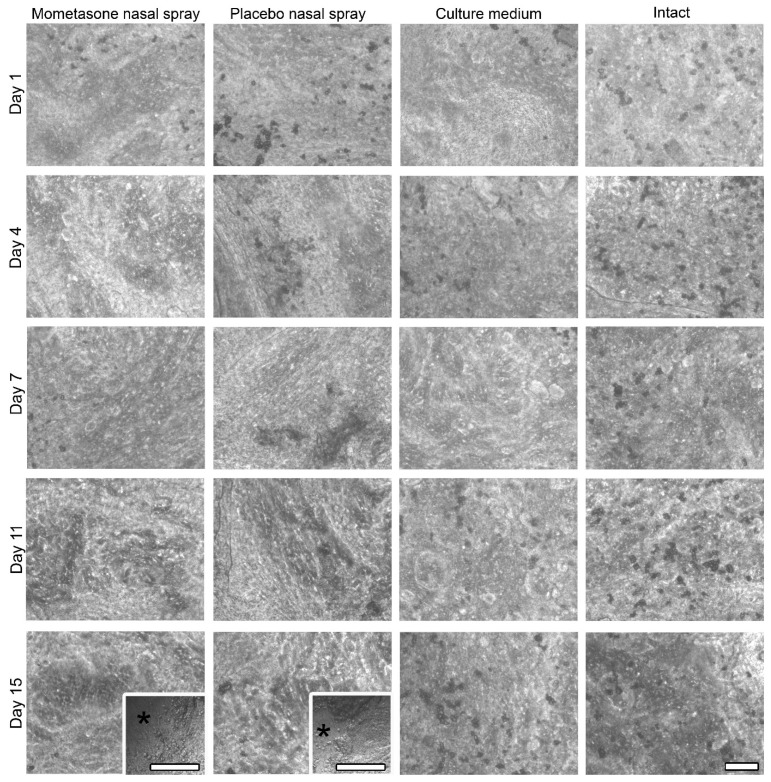
Morphological assessment of the nasal MucilAir™ in vitro models treated with 10-fold diluted mometasone or placebo nasal spray, culture medium treated or untreated for 14 consecutive days using phase-contrast microscopy. The apical surface view is shown. Cell shedding is observed only at the periphery in the mometasone- and placebo-treated nasal MucilAir™ in vitro models on the last 2 days of the experiment (inserts, day 15). Asterisks indicate areas without cells. Scale bars, 100 µm.

However, we observed cell shedding at the periphery of the mometasone- and placebo-treated nasal in vitro models on days 14 and 15. Conversely, cell shedding was not observed in the culture medium-treated and intact nasal in vitro models (Figure 5, Supplementary Figure S2).

### 3.4. LDH Cytotoxicity Analysis

**Figure 6:**
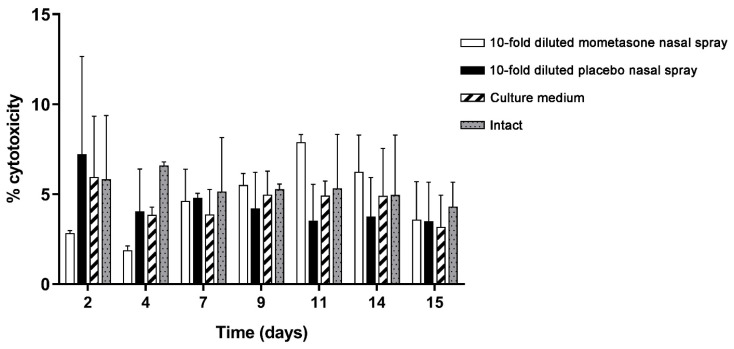
Lactate dehydrogenase (LDH) cytotoxicity assay of repeated doses of 10-fold diluted mometasone and placebo nasal spray, culture medium or intact condition on the nasal MucilAir™ in vitro model. The results are expressed as a percentage of cytotoxicity relative to the maximum LDH activity (of RPMI 2650 cells). Bars represent mean ± S.E. of two independent experiments. Two-way ANOVA with Tukey’s multiple comparisons test was performed. No statistical differences in cytotoxicity between the treated and untreated nasal in vitro models were observed.

The nasal MucilAir™ in vitro models were repeatedly exposed for 2 weeks to 10-fold diluted mometasone nasal spray, 10-fold diluted placebo nasal spray, or culture medium (controls) (Figure 6). Results were compared to the intact nasal MucilAir™ in vitro models (untreated cultures), which were analysed following the same protocol as the treated cultures. Cytotoxicity was very low in all nasal in vitro models (treated and untreated). Furthermore, 10-fold diluted mometasone or placebo nasal spray did not exhibit significantly different cytotoxicity compared to the controls (culture media-treated and intact cultures) (Figure 6, Supplementary Table S3). These results imply that the tested nasal sprays do not exert higher cytotoxicity than the culture media.

### 3.5. Post-Treatment Ultrastructural Analysis

**Figure 7:**
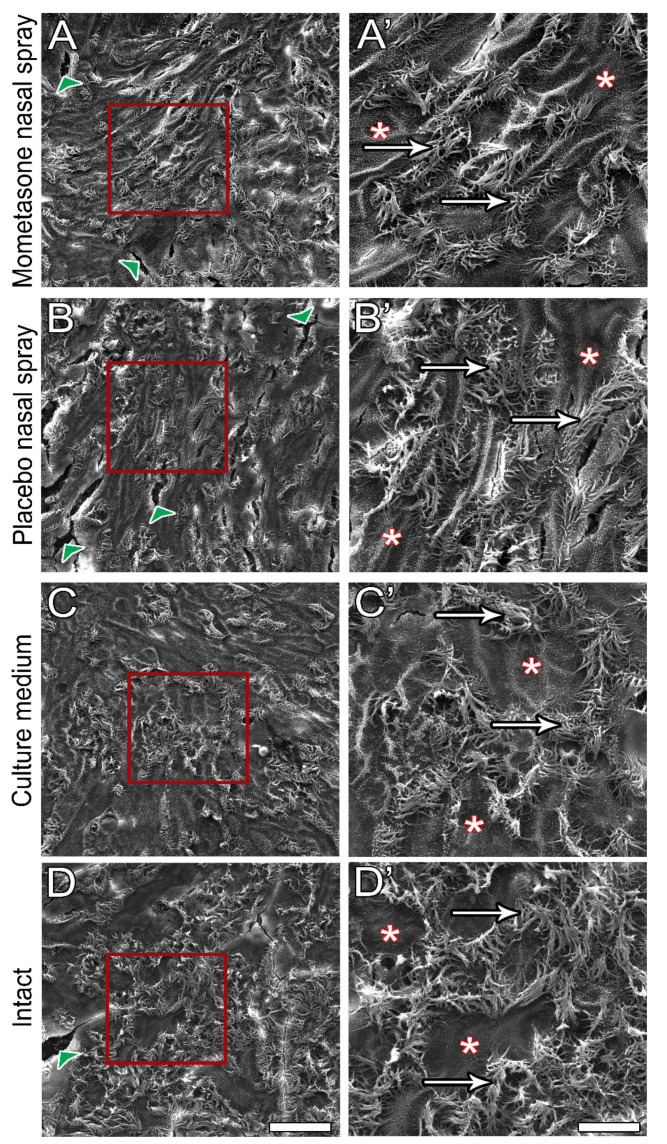
Post-treatment ultrastructural SEM analysis of the apical surface topography of the nasal MucilAir™ in vitro models. Cultures were treated with 10-fold diluted mometasone nasal spray (**A**,**A’**), 10-fold diluted placebo nasal spray (**B**,**B’**), or culture medium (**C**,**C’**) for 14 consecutive days. Intact cultures were not treated (**D**,**D’**). Ciliated cells (arrows) and non-ciliated cells (asterisks) with apparent cell boundaries are observed in all cultures, regardless of the treatment. Arrowheads indicate artefacts. The red framed areas in images (**A**–**D**) are magnified in (**A’**–**D’**). Scale bars, 50 µm (**A**–**D**) and 25 µm (**A’**–**D’**).

**Figure 8:**
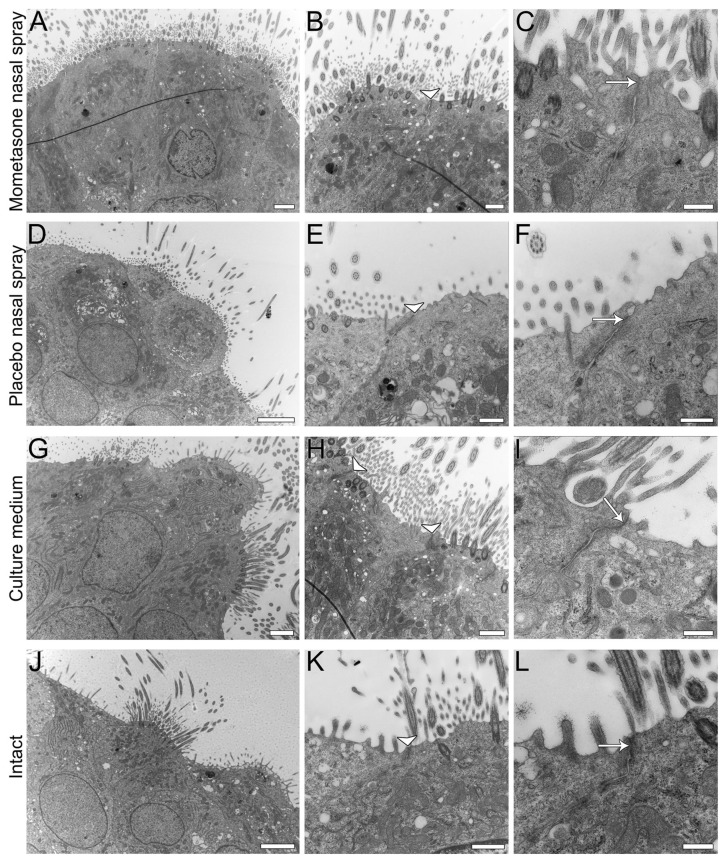
Post-treatment ultrastructural TEM analysis of the nasal MucilAir™ in vitro models. Cultures were treated with 10-fold diluted mometasone nasal spray (**A**–**C**), 10-fold diluted placebo nasal spray (**D**–**F**), or culture medium (**G**–**I**) for 14 consecutive days. Intact cultures were not treated (**J**–**L**). Ciliated and non-ciliated cells are connected with junctional complexes (arrowheads in **B**,**E**,**H**,**K**) in all MucilAir™ cultures (treated and untreated). Well-formed tight junctions are seen in higher magnifications (arrows in **C**,**F**,**I**,**L**). Scale bars, 6 µm (**D**), 4 µm (**J**), 2 µm (**A**,**G**), 1 µm (**B**,**E**,**H**,**K**), 600 nm (**F**), and 400 nm (**C**,**I**,**L**).

After 14 days of continuous daily treatment, the diluted mometasone- and placebo-treated nasal MucilAir™ in vitro models were analysed to examine potential nasal spray cytotoxicity. SEM (Figure 7) and TEM (Figure 8) analyses were performed. No significant differences in morphology, ultrastructure, or integrity were found between the control and nasal spray-treated nasal in vitro models. SEM analysis showed a similar number of ciliated cells with long cilia in both control (culture media-treated and intact cultures) and nasal spray-treated cultures (mometasone and placebo nasal spray, Figure 7). Furthermore, TEM analysis confirmed tissue integrity in all nasal models, i.e., most epithelial cells were tightly connected via tight junctions, regardless of the treatment (Figure 8). Occasionally, however, disrupted tight junctions were observed in both nasal sprays (mometasone and placebo)-treated and control nasal in vitro models (culture media-treated and untreated cultures). Cell shedding was also observed in control cultures but was less pronounced than in the mometasone- and placebo-treated nasal in vitro models (pronounced in the periphery, Figure 5, Supplementary Figure S2).

## 4. Discussion

Intranasal administration is a logical delivery choice for the topical treatment of local diseases of the nose and paranasal sinuses, such as allergic and non-allergic rhinitis and sinusitis. Nevertheless, the main purpose of nasal airways is to protect the sensitive lungs from dangerous exposures, and not to serve as an administration route for drugs and vaccines [2,47]. It is therefore essential that intranasal formulations are tested for their effects on the integrity of the primary innate nasal defence mechanism, i.e., mucociliary clearance, before use. The aim of this study was to assess the usefulness of the previously developed set-up for ciliotoxicity testing [34] to evaluate the cumulative effect of nasal sprays on ciliary activity in vitro after repeated exposure, as repeated doses of topical nasal formulations are usually used.

### 4.1. Applicability of the Nasal MucilAir™ In Vitro Model for the Analysis of Repeated Doses Ciliotoxicity and Cytotoxicity of Topical Nasal Formulations

The nasal MucilAir™ in vitro model is a fully differentiated pseudostratified epithelium cultured at the air-liquid interface that contains all the structural characteristics of nasal (respiratory) mucosa in vivo. Namely, the nasal in vitro models consist of basal, goblet, and ciliated cells connected by tight and adherent junctions (Figure 1, Figure 7 and Figure 8), which are expressed homogenously over the entire apical surface and enable the nasal in vitro model to form a polarized barrier [24]. The nasal MucilAir™ in vitro model display functional cilia (Video S1), mucus production, ion channel function, and secretion of cytokines, chemokines, and various proteases [48,49]. A crucial characteristic of the nasal MucilAir™ in vitro model is its usefulness for long-term chronic studies and repeated exposure, as it retains functional properties with ciliary beating over several months. Furthermore, cell growth on porous membranes allows morphological and ultrastructural analyses after treatment ([34], http://www.epithelix.com, accessed on 25 August 2021). The nasal MucilAir™ in vitro model has been used to study repeated exposure to smoke [50,51,52], gasoline engine emissions [53], formaldehyde [54], a novel hypertonic seawater solution for nasal lavage [55], and inhaled drugs or drug candidates [56]. To the best of our knowledge, our study is the first to investigate the relevance, value, and possible application of the nasal MucilAir™ in vitro model to predict chronic in vivo ciliotoxicity and cytotoxicity of topical nasal formulations. In addition, this is the first study to examine chronic nasal spray ciliotoxicity in human nasal epithelia in vitro.

### 4.2. The Importance of In Vitro Assessment of the Effect of Chronic Topical Nasal Formulation Exposure on CBF

Topical nasal drugs, such as antihistamines, corticosteroids, and decongestants, are commonly used to treat rhinitis, sinusitis, and related allergic or chronic conditions. This is due to their efficacy, tolerability, easy application, cost-effectiveness, and low risk of systemic side effects [1,2,3,4,5,6]. As topical nasal formulations are often repeatedly applied as long-term therapy, it is important to elucidate their chronic effects on mucociliary clearance and nasal mucosa. Ciliary beating is a driving force for mucociliary clearance. CBF is linearly correlated with mucociliary clearance and can thus be used to quantitatively determine mucociliary function [9]. Defective mucociliary clearance lengthen the contact times of the airways with pathogens, irritating or carcinogenic substances, which can results in respiratory infections and/or damaged mucosa [57]. Some studies have indicated that mucociliary transport or CBF are decreased in patients with chronic rhinosinusitis, allergic rhinitis, or sinusitis [58,59]. It is therefore of great importance to determine whether topical nasal drugs can hinder nasal ciliary activity.

The in vitro safety assessment of topically administered drugs based on CBF measurements has two important advantages. First, only in vitro experiments guarantee constant conditions and exclude factors that can influence CBF, such as stress, hormone secretion, inflammatory mediators, infections, osmolarity, pH, and temperature [15,60,61,62]. Second, the use of human nasal in vitro models overcomes the disadvantages of in vivo and ex vivo animal models with respect to species differences. Furthermore, the use of animals is reduced to a minimum in compliance with the guidelines for replacement, reduction, and refinement (3Rs) in laboratory animal use. By contrast, clinical trials are costly, time-consuming, and often burdensome on patients [27]; thus, they are not appropriate for screening a large number of formulation candidates. Initial in vitro screening assays for topical nasal drug formulation candidates represent a rapid, highly effective, and economical alternative to expensive clinical screenings in animals and humans.

However, in vitro experiments also have their limitations and cannot precisely duplicate in vivo conditions. Topical nasal formulations are distributed and diluted on the surface of nasal mucosa after application in vivo. Due to mucociliary transport, drug formulations can be removed from the natural environment in the nose. In addition, epithelial renewal continues in vivo [38]. Thus, in vitro models can lack a protective mucus layer and cannot entirely mimic the process of mucus removal from the nose, as the common technical set-up of cultured cells on inserts does not currently enable this. The dilution of nasal formulations by the produced mucus that is observed in vivo, however, can be adequately replicated also in vitro. Namely, a 5–10-fold dilution of nasal formulation has been proposed to mimic the in vivo situation and more realistically reflect the ciliotoxic effect of nasal formulations [46]. Jiao et al. [46] calculated that nasal products are diluted at least 5-fold after administration into the nose, as the healthy human nose contains approximately 0.4 mL of mucus, and spray doses are usually in the order of 0.1 mL in clinical practice. As mucus production can be increased in patients with allergic rhinitis, it is reasonable to test the nasal preparations at even greater dilutions. A 10-fold dilution of nasal spray was therefore analysed in our study in addition to undiluted nasal spray. In this way, a more realistic estimate of the drugs’ safety for in vivo use can be made. Substances that do not damage ciliated cells or affect mucociliary clearance in vitro can be considered safe for use in vivo [3].

### 4.3. The Observed Effects of Repeated Exposure to Clinically Relevant Doses of Mometasone Nasal Spray on CBF Are in Good Agreement with Those of In Vivo Studies

Mometasone furoate is a glucocorticosteroid used in nasal sprays as an effective topical medical treatment for seasonal or perennial allergic rhinitis and nasal polyposis [63]. The topical application of mometasone furoate as a nasal spray prevents high systemic concentrations and does not cause clinically significant adverse effects. A clinical trial on patients with allergic rhinitis showed positive results with significant symptom improvement [64,65]. Its clinical efficacy combined with a favourable safety and tolerability profile confers its favourable benefit-risk ratio [66]. Naclerio et al. [67] reported that a 2-week treatment with mometasone nasal spray (Nasonex, containing mometasone furoate and BKC) did not impair mucociliary clearance. Similarly, Pata et al. [68] showed that mometasone furoate nasal spray does not affect mucociliary clearance in patients with perennial allergic rhinitis.

In this study, we have shown that the effect of repeated treatment with mometasone nasal spray Mommox^®^ on CBF in the nasal MucilAir™ in vitro model is dose- and time-dependent (Figure 2, Figure 3, Figure 4 and Figure 5). Undiluted mometasone nasal spray treatment had a cumulative inhibitory effect on CBF and was cytotoxic, whereas a clinically relevant dose of Mommox^®^ spray showed no ciliotoxicity or cytotoxicity. Furthermore, the results obtained with a clinically relevant dose of Mommox^®^ nasal spray, which contains BKC (Figure 4, Figure 5, Figure 6, Figure 7 and Figure 8), are consistent with the results of clinical studies on Nasonex mometasone nasal spray, which also contains BKC. Nasonex does not impair mucociliary clearance [67] and does not damage nasal mucosa [69]. Moreover, a clinical study has shown that Mommox^®^ nasal spray is effective and well-tolerated in the treatment of seasonal allergic rhinitis [64]. Furthermore, we have also demonstrated that repeated treatment with placebo nasal spray at an undiluted concentration exerts cilio-inhibiting and cytotoxic effects (Figure 2 and Figure 3). Conversely, at clinically relevant doses, it did not exhibit ciliotoxicity (Figure 4, Figure 5, Figure 6, Figure 7 and Figure 8). Since placebo spray contains excipients without the active drug, we speculate that mometasone furoate itself has no adverse effects on ciliary activity and is not cytotoxic. By contrast, BKC, a preservative in nasal sprays, has been shown to inhibit ciliary activity and damage nasal epithelial cells in vitro [34,70,71]. It is therefore possible that the cumulative cilio-inhibitory and cytotoxic effects of undiluted nasal sprays (Mommox^®^ and placebo) could be due to BKC. However, 10-fold diluted Mommox^®^ and placebo did not affect ciliary function and were not cytotoxic. This is consistent with in vivo studies that showed that the toxic effect of BKC in vivo is inactivated by proteins in nasal secretions [33,72].

Interestingly, chronic treatment with culture media exhibited a cilio-stimulatory effect, possibly due to extensive mucus dilution and rinsing. Moreover, it is possible that culture media exerts similar cilio-stimulatory effects as isotonic saline, Ringer Lactate solution, isotonic seawater, etc., which have been shown to increase mucociliary clearance and CBF. These nasal irrigation solutions contain high levels of minerals and trace elements, such as calcium, potassium, magnesium, and zinc ions, which can assist in epithelial wound repair and ciliary beat regulation [3,73,74,75,76].

The existing data in the literature suggest that mucociliary clearance of the entire nasal mucosa occurs in as little as 10 min [77,78] to 20 min [79], while 30 min is considered the cut-off point for distinguishing between normal and impaired nasal mucociliary clearance [79]. In our study, 30 min of daily exposure to nasal sprays was implemented to more closely represent the impaired mucociliary clearance time observed in patients [80]. The results of our study suggest that clinically relevant doses of Mommox^®^ nasal spray can be considered safe, as even prolonged exposure (30 min versus 10 min) did not result in ciliotoxicity.

Taken together, our results demonstrate high sensitivity of the in vitro CBF analysis set-up in terms of dosing and exposure (repetitions) to topical nasal formulations. Furthermore, there is a good correlation between clinical in vivo and in vitro studies when nasal formulations are appropriately diluted to mimic in vivo conditions. This suggests the usefulness of the described CBF analysis set-up for safety studies regarding repeated exposure.

However, it is important to emphasize the potential limitations to in vitro chronic exposure studies. Ultrastructural analysis of the control nasal in vitro models (culture media-treated and intact cultures) showed that long-term experiments affect the integrity of the nasal MucilAir™ cultures. Namely, tight junctions were occasionally disrupted, and cell shedding occurred in certain regions by day 15. Cell shedding was also observed in the nasal spray-treated cultures by day 15 (Figure 5), and we suspect that further treatment and CBF measurements would lead to more extensive cell shedding. Furthermore, the disrupted tight junctions indicate that further treatment and measurements would also lead to more extensive cell detachment. It is therefore possible that chronic in vitro studies using topical nasal formulations and the nasal MucilAir™ nasal in vitro models may be limited to a period of 2 weeks. However, it is also possible that the reduced integrity of the nasal in vitro models may be due to aging (cell senescence).

## 5. Conclusions

Topical nasal drug formulations are recognized as a first-line treatment for local diseases of the nose and paranasal sinuses. Available data suggest that some active drugs and excipients of nasal formulations impede ciliary function and mucociliary clearance. It is therefore important to assess the effects of intranasal drugs on mucociliary functions before employing them to treat various nasal diseases. In vitro CBF assessment has been shown to be an accurate and reproducible technique. Topical nasal formulations are usually applied as long-term therapy with repeated doses; therefore, it is important to elucidate their chronic versus acute effect on mucociliary clearance and nasal mucosa. Our current study assessed an in vitro assay with clinically relevant doses of nasal formulations intended for chronic treatment and the 3D nasal MucilAir™ in vitro model, and our results correlate well with those of in vivo studies. This indicates that such an in vitro assay has great potential for topical nasal drug screening and may contribute to the 3Rs approach to animal testing.

## Data Availability

The authors declare that they have no known competing financial interests or personal relationships that could have appeared to influence the work reported in this paper.

## Supplementary Materials

The following are available online at https://www.mdpi.com/article/10.3390/pharmaceutics13111750/s1, Figure S1: Morphological assessment of the nasal MucilAirTM in vitro models treated with placebo nasal spray, undiluted mometasone nasal spray, and culture media for 10 consecutive days using stereoscopic microscopy, Scale bar, 100 µm, Figure S2: Morphological assessment of the nasal MucilAirTM in vitro models treated with 10-fold diluted mometasone and placebo nasal spray and culture media for 14 consecutive days using stereoscopic microscopy, Scale bar, 100 µm, Table S1: Results of two-way ANOVA analysis with Tukey’s multiple comparisons test of repeated exposure to undiluted nasal spray, Table S2: Results of two-way ANOVA analysis with Tukey’s multiple comparisons test of repeated exposure to diluted nasal spray, Table S3: Results of two-way ANOVA analysis with Tukey’s multiple comparisons test of LDH cytotoxicity, Video S1: Baseline CBF, Video S2: Undiluted Mommox—day 1, Video S3: Undiluted Mommox—day 7, Video S4: Undiluted Mommox—day 9, Video S5: Undiluted Placebo—day 1, Video S6: Undiluted Placebo—day 7, Video S7: Undiluted Placebo—day 11, Video S8: Culture medium—day 1, Video S9: Culture medium—day 11, Video S10: Intact—day 1, Video S11: Intact—day 7, Video S12: Diluted Mommox—day 1, Video S13: Diluted Mommox—day 7, Video S14: Diluted Mommox—day 15, Video S15: Diluted Placebo—day 1, Video S16: Diluted Placebo—day 7, Video S17: Diluted Placebo—day 15, Video S18: Culture medium—day 1, Video S19: Culture medium—day 9, Video S20: Culture medium—day 14, Video S21: Intact—day 1, Video S22: Intact—day 7, Video S23: Intact—day 14.
