# Supplementary material for: In Vitro Ciliotoxicity and Cytotoxicity Testing of Repeated Chronic Exposure to Topical Nasal Formulations for Safety Studies
Source: Pharmaceutics. 2021 Oct 20;13(11):1750. doi: 10.3390/pharmaceutics13111750 (PMC8618987; doi:10.3390/pharmaceutics13111750)
Supplement: Supplementary file 1 [file pharmaceutics-13-01750-s001.zip › Supplementary Material/Supplementary Materials PROOFS_MEK.pdf]

pharmaceutics

Supplementary Materials: In Vitro Ciliotoxicity and Cytotoxicity Testing of Repeated Chronic Exposure to Topical Nasal Formulations for Safety Studies

Larisa Tratnjek, Nadica Sibinovska, Katja Kristan and Mateja Erdani Kreft

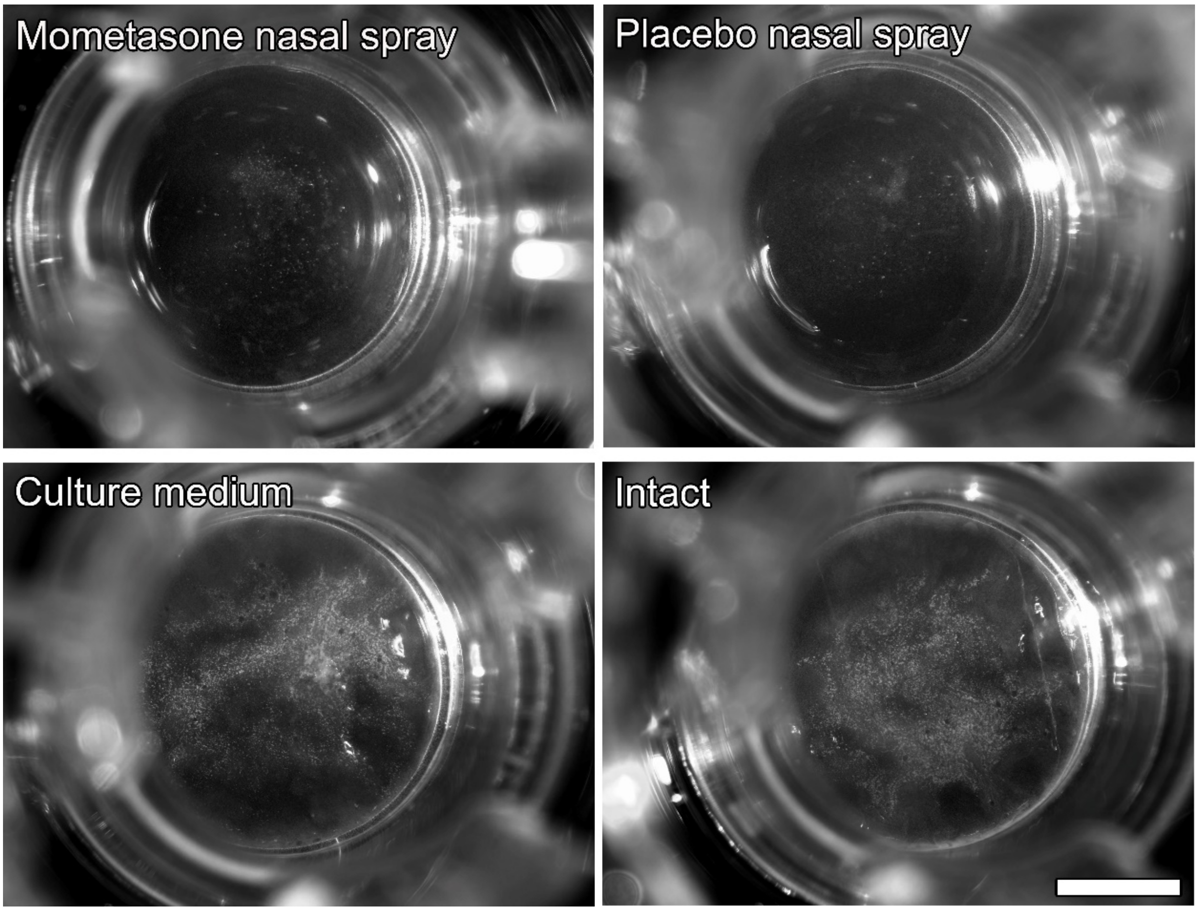

Figure S1. Morphological assessment of the nasal MucilAir™ in vitro models treated with placebo nasal spray, undiluted mometasone nasal spray, and culture media for 10 consecutive days using stereoscopic microscopy. The apical surface views of nasal models on day 11 are shown. Almost complete cell detachment is observed in the undiluted mometasone- and placebo spray-treated nasal MucilAir™ in vitro models in contrast to culture medium-treated and intact cultures without observed cell shedding. Scale bar, 100 µm.

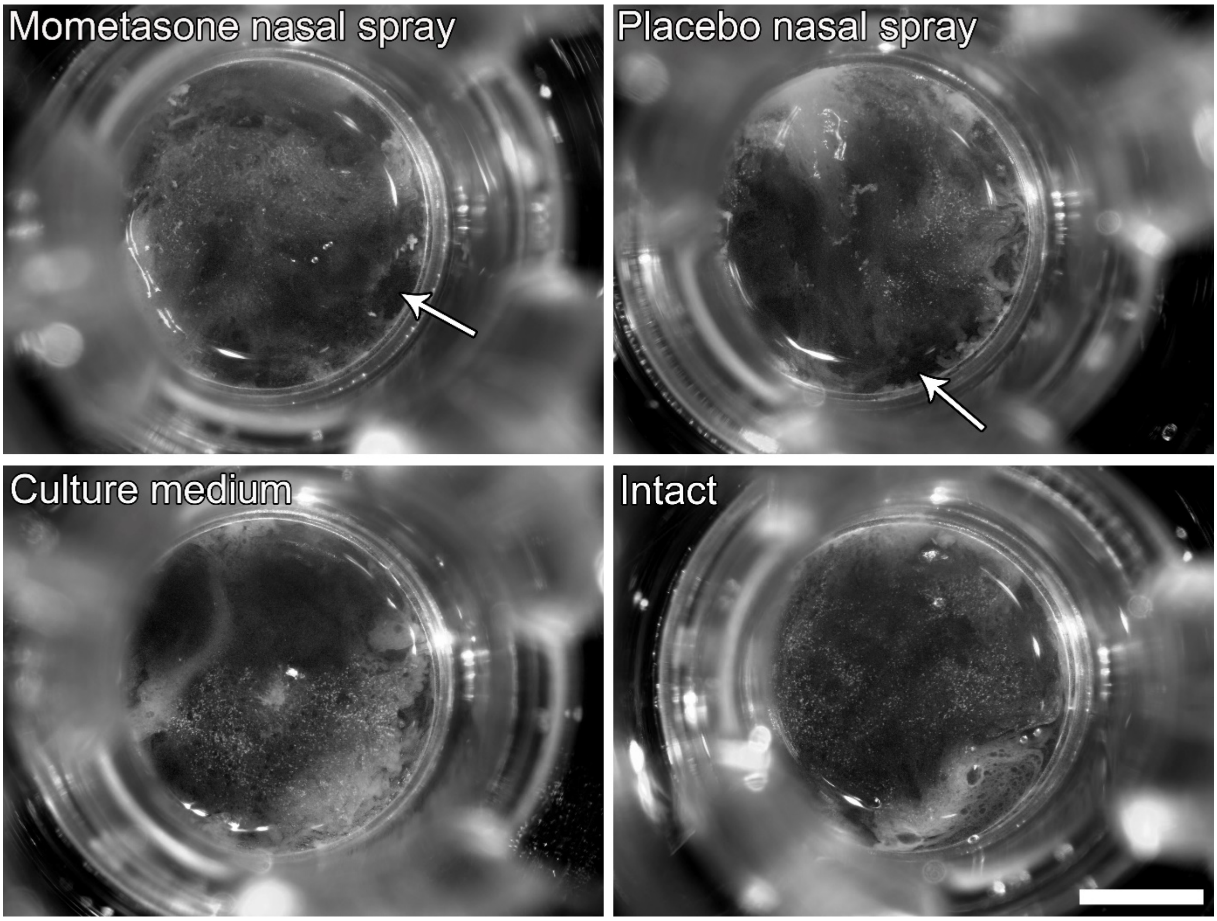

Figure S2. Morphological assessment of the nasal MucilAir™ in vitro models treated with 10-fold diluted mometasone and placebo nasal spray, and culture media for 14 consecutive days using stereoscopic microscopy. The apical surface views of nasal models on day 14 are shown. On the last two days of the 2 weeks' experiment cell detachment is observed in peripheral areas in mometasone- and placebo spray-treated the nasal MucilAir™ in vitro models (arrows) in the contrast to culture medium-treated and intact nasal MucilAir™ in vitro models that do not show any cell detachment. Scale bar, 100 μm.

Table S1. Results of two-way ANOVA analysis with Tukey's multiple comparisons test of repeated exposure to undiluted investigational nasal spray' experimental data

2way ANOVA Multiple comparisons					
**Within each row, compare columns (simple effects within rows)**					
**Number of families**	**4**				
**Number of comparisons per family**	**6**				
**Alpha**	**0.05**				
**Tukey's multiple comparisons test**	**Mean Difference**	**95% Confidence Interval of difference**	**Significant**	**Summary**	**Adjusted P Value**
Day 1					
Mommox vs. Placebo	0.001880	−23.12 to 23.12	No	ns	> 0.9999
Mommox vs. Culture medium	0.009360	−23.11 to 23.13	No	ns	> 0.9999
Mommox vs. Intact	−2.054	−25.18 to 21.07	No	ns	0.9958
Placebo vs. Culture medium	0.007480	−23.11 to 23.13	No	ns	> 0.9999
Placebo vs. Intact	−2.056	−25.18 to 21.07	No	ns	0.9957
Culture medium vs. Intact	−2.063	−25.19 to 21.06	No	ns	0.9957
Day 2					
Mommox vs. Placebo	−27.79	−50.91 to −4.670	Yes	*	0.0111
Mommox vs. Culture medium	−28.70	−51.82 to −5.579	Yes	**	0.0080
Mommox vs. Intact	−39.43	−62.55 to −16.31	Yes	****	< 0.0001
Placebo vs. Culture medium	−0.9093	−24.03 to 22.21	No	ns	0.9996
Placebo vs. Intact	−11.64	−34.76 to 11.48	No	ns	0.5642
Culture medium vs. Intact	−10.73	−33.85 to 12.39	No	ns	0.6288
Day 4					
Mommox vs. Placebo	−21.70	−44.83 to 1.417	No	ns	0.0746
Mommox vs. Culture medium	−16.34	−39.46 to 6.787	No	ns	0.2641
Mommox vs. Intact	−28.13	−51.25 to -5.003	Yes	**	0.0098
Placebo vs. Culture medium	5.370	−17.75 to 28.49	No	ns	0.9323
Placebo vs. Intact	−6.421	−29.54 to 16.70	No	ns	0.8905
Culture medium vs. Intact	−11.79	−34.91 to 11.33	No	ns	0.5534
Day 7					
Mommox vs. Placebo	−17.62	−40.74 to 5.507	No	ns	0.2028
Mommox vs. Culture medium	−59.81	−82.93 to −36.69	Yes	****	< 0.0001
Mommox vs. Intact	−92.34	−115.5 to −69.22	Yes	****	< 0.0001
Placebo vs. Culture medium	−42.20	−65.32 to −19.07	Yes	****	< 0.0001
Placebo vs. Intact	−74.72	−97.85 to −51.60	Yes	****	< 0.0001
Culture medium vs. Intact	−32.53	−55.65 to −9.405	Yes	**	0.0018

Analysis was performed with the Graph Pad Prism software. ns: not significant; *: p-value <0.05, **: p-value <0.01. ****: p-value <0.0001 significant

Table S2. Results of two-way ANOVA analysis with Tukey's multiple comparisons test of repeated exposure to diluted investigational nasal spray' experimental data.

2way ANOVA Multiple comparisons					
**Within each row, compare columns (simple effects within rows)**					
**Number of families**		**8**			
**Number of comparisons per family**		**6**			
**Alpha**		**0.05**			
**Tukey's multiple comparisons test**	Mean Difference	95% Confidence Interval of difference	Significant	Summary	Adjusted P Value
Mommox vs. Placebo	−4.654e-006	−14.14 to 14.14	No	ns	> 0.9999
Mommox vs. Culture medium	3.662e-006	−14.14 to 14.14	No	ns	> 0.9999
Mommox vs. Intact	−4.082e-006	−14.14 to 14.14	No	ns	> 0.9999
Placebo vs. Culture medium	8.316e-006	−14.14 to 14.14	No	ns	> 0.9999
Placebo vs. Intact	5.722e-007	−14.14 to 14.14	No	ns	> 0.9999
Culture medium vs. Intact	−7.744e-006	−14.14 to 14.14	No	ns	> 0.9999
Day 2					
Mommox vs. Placebo	−11.28	−25.42 to 2.866	No	ns	0.1701
Mommox vs. Culture medium	−1.896	−16.04 to 12.24	No	ns	0.9859
Mommox vs. Intact	4.919	−9.222 to 19.06	No	ns	0.8076
Placebo vs. Culture medium	9.379	−4.762 to 23.52	No	ns	0.3210
Placebo vs. Intact	16.19	2.054 to 30.34	Yes	*	0.0172
Culture medium vs. Intact	6.816	−7.325 to 20.96	No	ns	0.6017
Day 4					
Mommox vs. Placebo	0.5616	−13.58 to 14.70	No	ns	0.9996
Mommox vs. Culture medium	3.896	−10.25 to 18.04	No	ns	0.8938
Mommox vs. Intact	10.27	−3.866 to 24.42	No	ns	0.2420
Placebo vs. Culture medium	3.334	−10.81 to 17.48	No	ns	0.9301
Placebo vs. Intact	9.713	−4.428 to 23.85	No	ns	0.2899
Culture medium vs. Intact	6.379	−7.762 to 20.52	No	ns	0.6521
Day 7					
Mommox vs. Placebo	−3.800	−17.94 to 10.34	No	ns	0.9005
Mommox vs. Culture medium	−8.356	−22.50 to 5.785	No	ns	0.4258
Mommox vs. Intact	1.094	−13.05 to 15.23	No	ns	0.9972
Placebo vs. Culture medium	−4.556	−18.70 to 9.584	No	ns	0.8409
Placebo vs. Intact	4.894	−9.247 to 19.03	No	ns	0.8101
Culture medium vs. Intact	9.450	−4.691 to 23.59	No	ns	0.3142
Day 9					
Mommox vs. Placebo	−8.072	−22.21 to 6.069	No	ns	0.4572
Mommox vs. Culture medium	−16.79	−30.93 to −2.648	Yes	*	0.0123
Mommox vs. Intact	−0.4732	−14.61 to 13.67	No	ns	0.9998
Placebo vs. Culture medium	−8.717	−22.86 to 5.424	No	ns	0.3873
Placebo vs. Intact	7.598	−6.542 to 21.74	No	ns	0.5109
Culture medium vs. Intact	16.32	2.175 to 30.46	Yes	*	0.0161
Day 11					
Mommox vs. Placebo	5.437	−8.704 to 19.58	No	ns	0.7559

Mommox vs. Culture medium	−0.1705	−14.31 to 13.97	No	ns	> 0.9999
Mommox vs. Intact	−2.743	−16.88 to 11.40	No	ns	0.9593
Placebo vs. Culture medium	−5.607	−19.75 to 8.534	No	ns	0.7379
Placebo vs. Intact	−8.180	−22.32 to 5.961	No	ns	0.4452
Culture medium vs. Intact	−2.573	−16.71 to 11.57	No	ns	0.9661
Day 14					
Mommox vs. Placebo	−8.226	−22.37 to 5.915	No	ns	0.4401
Mommox vs. Culture medium	−15.74	−29.88 to −1.594	Yes	*	0.0222
Mommox vs. Intact	−7.269	−21.41 to 6.872	No	ns	0.5490
Placebo vs. Culture medium	−7.509	−21.65 to 6.632	No	ns	0.5212
Placebo vs. Intact	0.9572	−13.18 to 15.10	No	ns	0.9981
Culture medium vs. Intact	8.466	−5.675 to 22.61	No	ns	0.4139
Day 15					
Mommox vs. Placebo	21.27	7.129 to 35.41	Yes	***	0.0007
Mommox vs. Culture medium	5.181	−8.960 to 19.32	No	ns	0.7821
Mommox vs. Intact	13.70	−0.4449 to 27.84	No	ns	0.0617
Placebo vs. Culture medium	−16.09	−30.23 to −1.948	Yes	*	0.0183
Placebo vs. Intact	−7.574	−21.71 to 6.567	No	ns	0.5137
Culture medium vs. Intact	8.515	−5.625 to 22.66	No	ns	0.4086

Analysis was performed with the Graph Pad Prism software. ns: not significant; *: p-value <0.05, ***: p-value <0.001

Table S3. Results of two-way ANOVA analysis with Tukey's multiple comparisons test of LDH cytotoxicity analysis' experimental data.

2way ANOVA Multiple comparisons					
**Within each row, compare columns (simple effects within rows)**					
**Number of families**			**7**		
**Number of comparisons per family**			**6**		
**Alpha**			**0.05**		
**Tukey's multiple comparisons test**	**Mean Difference**	**95.00% Confidence Interval of difference**	**Below threshold**	**Summary**	**Adjusted P Value**
**Day 2 (24 h)**					
**Mometasone spray vs. Placebo nasal spray**	4.395	−4.062 to 12.85	No	ns	0.4985
**Mometasone spray vs. Culture medium**	1.270	−7.187 to 9.727	No	ns	0.9763
**Mometasone spray vs. Intact**	1.395	−7.062 to 9.852	No	ns	0.9690
**Placebo nasal spray vs. Culture medium**	−3.125	−11.58 to 5.332	No	ns	0.7456
**Placebo nasal spray vs. Intact**	−3.000	−11.46 to 5.457	No	ns	0.7682
**Culture medium vs. Intact**	0.1250	−8.332 to 8.582	No	ns	>0.9999
**Day 4 (72 h)**					
**Mometasone spray vs. Placebo nasal spray**	2.150	−6.307 to 10.61	No	ns	0.8985
**Mometasone spray vs. Culture medium**	0.1800	−8.277 to 8.637	No	ns	>0.9999
**Mometasone spray vs. Intact**	−2.570	−11.03 to 5.887	No	ns	0.8399
**Placebo nasal spray vs. Culture medium**	−1.970	−10.43 to 6.487	No	ns	0.9195
**Placebo nasal spray vs. Intact**	−4.720	−13.18 to 3.737	No	ns	0.4373
**Culture medium vs. Intact**	−2.750	−11.21 to 5.707	No	ns	0.8111
**Day 7 (144 h)**					
**Mometasone spray vs. Placebo nasal spray**	0.1500	−8.307 to 8.607	No	ns	>0.9999
**Mometasone spray vs. Culture medium**	0.9150	−7.542 to 9.372	No	ns	0.9908
**Mometasone spray vs. Intact**	−0.3650	−8.822 to 8.092	No	ns	0.9994
**Placebo nasal spray vs. Culture medium**	0.7650	−7.692 to 9.222	No	ns	0.9946
**Placebo nasal spray vs. Intact**	−0.5150	−8.972 to 7.942	No	ns	0.9983
**Culture medium vs. Intact**	−1.280	−9.737 to 7.177	No	ns	0.9757
**Day 9 (192 h)**					
**Mometasone spray vs. Placebo nasal spray**	−1.295	−9.752 to 7.162	No	ns	0.9749
**Mometasone spray vs. Culture medium**	−0.7600	−9.217 to 7.697	No	ns	0.9947
**Mometasone spray vs. Intact**	−1.070	−9.527 to 7.387	No	ns	0.9855
**Placebo nasal spray vs. Culture medium**	0.5350	−7.922 to 8.992	No	ns	0.9981

**Placebo nasal spray vs. Intact**	0.2250	−8.232 to 8.682	No	ns	0.9999
**Culture medium vs. Intact**	−0.3100	−8.767 to 8.147	No	ns	0.9996
**Day 11 (240 h)**					
**Mometasone spray vs. Placebo nasal spray**	−4.355	−12.81 to 4.102	No	ns	0.5062
**Mometasone spray vs. Culture medium**	−1.390	−9.847 to 7.067	No	ns	0.9693
**Mometasone spray vs. Intact**	−1.790	−10.25 to 6.667	No	ns	0.9379
**Placebo nasal spray vs. Culture medium**	2.965	−5.492 to 11.42	No	ns	0.7744
**Placebo nasal spray vs. Intact**	2.565	−5.892 to 11.02	No	ns	0.8407
**Culture medium vs. Intact**	−0.4000	−8.857 to 8.057	No	ns	0.9992
**Day 14 (312 h)**					
**Mometasone spray vs. Placebo nasal spray**	−2.500	−10.96 to 5.957	No	ns	0.8505
**Mometasone spray vs. Culture medium**	−1.160	−9.617 to 7.297	No	ns	0.9817
**Mometasone spray vs. Intact**	−1.205	−9.662 to 7.252	No	ns	0.9796
**Placebo nasal spray vs. Culture medium**	1.340	−7.117 to 9.797	No	ns	0.9724
**Placebo nasal spray vs. Intact**	1.295	−7.162 to 9.752	No	ns	0.9749
**Culture medium vs. Intact**	−0.04500	−8.502 to 8.412	No	ns	>0.9999
**Day 15 (336 h)**					
**Mometasone spray vs. Placebo nasal spray**	−0.08500	−8.542 to 8.372	No	ns	>0.9999
**Mometasone spray vs. Culture medium**	0.3150	−8.142 to 8.772	No	ns	0.9996
**Mometasone spray vs. Intact**	−0.8150	−9.272 to 7.642	No	ns	0.9935
**Placebo nasal spray vs. Culture medium**	0.4000	−8.057 to 8.857	No	ns	0.9992
**Placebo nasal spray vs. Intact**	−0.7300	−9.187 to 7.727	No	ns	0.9953
**Culture medium vs. Intact**	−1.130	−9.587 to 7.327	No	ns	0.9831

Analysis was performed with the Graph Pad Prism software. ns: not significant.
